# Synchronous Triple Malignancy Presentation of Multiple Myeloma and Prefibrotic Primary Myelofibrosis in a Patient With Mucinous Breast Carcinoma: A Case Report From Rural South Africa

**DOI:** 10.1155/crh/7459223

**Published:** 2026-09-24

**Authors:** Michael A. Linström, Dean C. Mitchell, Riyaadh Roberts, Robert K. Lohlun

**Affiliations:** ^1^ Division of Haematological Pathology, Department of Pathology, Stellenbosch University, Cape Town, South Africa, sun.ac.za; ^2^ Haematology Laboratory, National Health Laboratory Services, Green Point, Cape Town, South Africa, nhls.ac.za; ^3^ Department of Internal Medicine, George Regional Hospital, George, South Africa; ^4^ Division of Anatomical Pathology, Department of Pathology, University of Cape Town, Cape Town, South Africa, uct.ac.za; ^5^ Division of Anatomical Pathology, Groote Schuur Hospital, National Health Laboratory Services, Cape Town, South Africa, nhls.ac.za; ^6^ Division of Haematology, Department of Pathology, University of Cape Town, Cape Town, South Africa, uct.ac.za; ^7^ Division of Haematology, Groote Schuur Hospital, National Health Laboratory Services, Cape Town, South Africa, nhls.ac.za

**Keywords:** case report, mucinous breast carcinoma, multiple myeloma, multiple primary neoplasms, primary myelofibrosis, synchronous

## Abstract

**Background:**

Multiple primary malignant neoplasms (MPMNs) diagnosed synchronously are rare, with an incidence of less than 1%. The occurrence of triple synchronous malignancies is exceedingly uncommon, and to our knowledge, no prior reports describe the concurrent presentation of a myeloproliferative neoplasm, plasma cell dyscrasia and breast cancer. Such cases pose considerable diagnostic and therapeutic challenges, particularly in settings with limited access to advanced molecular testing and oncologic therapies.

**Case Presentation:**

A 67‐year‐old woman presented with a pathological femoral fracture, marked leucocytosis and thrombocytosis. Histological examination of the fracture site revealed a kappa‐restricted plasmacytoma meeting diagnostic criteria for multiple myeloma (R‐ISS Stage 3). Bone marrow biopsy demonstrated hypercellularity with atypical clustered megakaryocytes, consistent with prefibrotic primary myelofibrosis, and molecular analysis confirmed a *JAK2 V617F* mutation. During evaluation, a right breast mass was confirmed on core biopsy as an invasive carcinoma with focal mucinous differentiation (ER/PR‐positive, HER2‐negative and Ki‐67 25%), consistent with a luminal B–like profile. The patient commenced therapy with melphalan and prednisone for myeloma and anastrozole for breast carcinoma, with multidisciplinary input guiding treatment sequencing.

**Discussion:**

This case represents an exceptionally rare triad of synchronous malignancies spanning myeloid, plasma‐cell and epithelial lineages. While myeloproliferative neoplasms are associated with an elevated risk of secondary neoplasia, the simultaneous occurrence of three independent malignancies raises the possibility of shared pathogenic mechanisms such as clonal haematopoiesis or an underlying germline predisposition. The presentation highlights the diagnostic complexity of distinguishing independent primaries from metastatic disease and illustrates the importance of maintaining diagnostic vigilance. Limited access to next‐generation sequencing and novel therapies further complicates management in resource‐constrained healthcare environments.

**Conclusion:**

Triple synchronous primary malignancies are exceedingly rare and diagnostically challenging. This case emphasises the necessity of a systematic, multidisciplinary approach to evaluation and management, even in low‐resource settings, to ensure accurate diagnosis and optimal coordination of care across multiple oncologic pathologies.

## 1. Introduction

Multiple primary malignant neoplasms (MPMNs) diagnosed at or near presentation are uncommon, with overall reported incidences ranging from 0.2% to 0.8% depending on cancer type, study population and follow‐up period [1–4]. Synchronous MPMNs are most often defined as those diagnosed within 6 months of the index malignancy, although some studies use a shorter 2‐month interval [5–7]. Triple synchronous MPMNs are exceedingly rare, with only isolated case reports and small series available, and no robust incidence estimates from population‐based datasets [8, 9]. The co‐occurrence of breast carcinoma with either a myeloproliferative neoplasm (MPN) or a plasma cell dyscrasia has been described, though each association remains uncommon [10, 11]. The recognition of synchronous MPMN presents significant diagnostic and therapeutic challenges, including distinguishing metastases from independent primaries, maintaining vigilance for additional occult malignancies and sequencing treatment strategies. To our knowledge, there are no prior reports of synchronous presentation of breast cancer, a MPN and a plasma cell dyscrasia. We present what we believe to be the first such reported case.

## 2. Case Report

**FIGURE 1:**
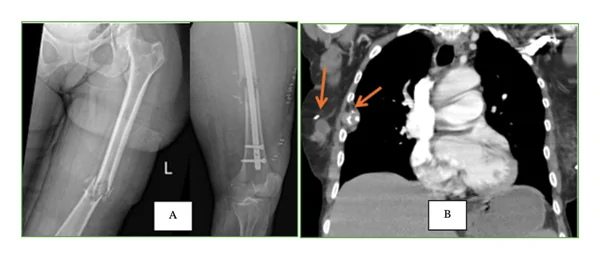
(A) X‐ray of the left femur midshaft diaphyseal compound fracture pre‐ and postsurgery. (B) Computed tomography of the chest with an expansile lytic lesion of the right 5th rib and a right breast mass.

A 67‐year‐old woman with a history of essential hypertension, previous pulmonary tuberculosis, chronic iron deficiency anaemia of uncertain aetiology and a 30‐pack‐year smoking history presented to Beaufort‐West Hospital in rural South Africa (February 2025) with a pathological midshaft fracture of the left femur following a low‐energy fall (Figure 1). Testing for human immunodeficiency virus (HIV) was negative.

Initial laboratory investigations revealed a neutrophil‐predominant leucocytosis (white cell count 44.2 × 10^9^/L), marked thrombocytosis (platelets 1478 × 10^9^/L) and mild microcytic hypochromic anaemia (haemoglobin 10 g/dL; mean corpuscular volume 67.8 fL; mean corpuscular haemoglobin 18.9 pg). Serum protein electrophoresis demonstrated an IgG kappa monoclonal protein with a gamma‐region peak of 6 g/L, with a mildly elevated kappa:lambda ratio. Serum calcium and renal function were within normal limits. Serum lactate dehydrogenase and serum beta‐2‐microglobulin were elevated (691 U/L and 5.9 mg/L, respectively), with a low serum albumin (27 g/L). Osteolytic bone lesions of the right fifth and left sixth rib were discovered using computed tomographic (CT) imaging (Figure 1).

**FIGURE 2:**
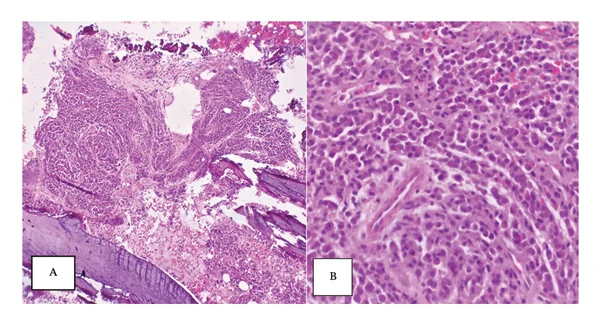
Bone fragment histology of the right femur fracture: (A) fibrous fragments with monotypic cellular infiltrate (H&E × 10); (B) dense clusters of mature plasma cells (H&E × 40).

She was referred to a regional hospital, where the fracture was stabilised with an intramedullary nail (Figure 1). Histopathological examination of an intraoperative bone specimen revealed fibrous tissue fragments infiltrated by small, mature plasma cells demonstrating kappa light chain restriction (Figure 2), consistent with a plasmacytoma. The plasmacytoma together with the associated end‐organ damage, namely, lytic bone lesions, meet the criteria for multiple myeloma (MM) [12].

Fluorescence in situ hybridisation (FISH) was performed on the plasmacytoma sample and was negative for high‐risk cytogenetic abnormalities, including del (1p), gain (1q), del (17p) and IGH (14q32) rearrangements [13]. The sB2M > 5.5 mg/L together with the LDH above the upper limit of normal places the patient at Stage 3 using the revised international staging system (R‐ISS) for MM [14].

Review of prior medical records revealed that the leucocytosis and thrombocytosis had been persistent since 2018. On examination, there was no palpable splenomegaly, and the patient reported no history of venous thromboembolism at unusual sites.

**FIGURE 3:**
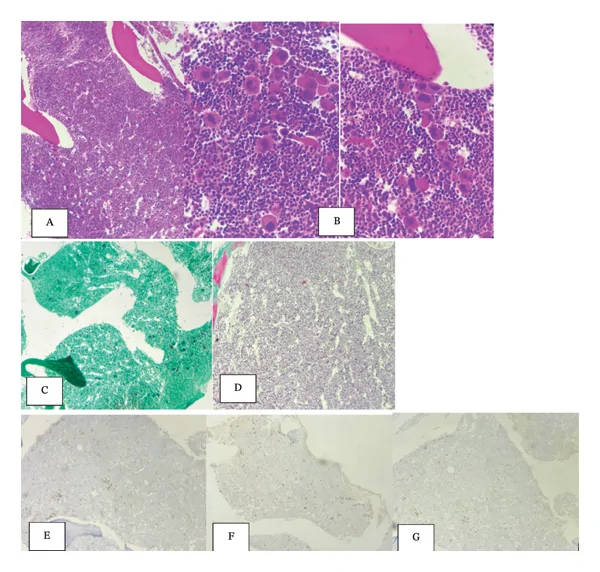
Bone marrow biopsy of the right posterior iliac spine. (A) Hypercellular marrow with increased vascularity and prominent megakaryocyte proliferation (H&E × 10). (B) Small and large clusters of variable atypical megakaryocytes with endosteal translocation (H&E × 40). (C) Normal scattered linear reticulin, grade MF‐0 (reticulin stain × 10). (D) Absence of intertrabecular collagen deposition (Masson’s trichrome × 10). (E) Normal plasma cell number and distribution (CD138 IHC × 10). (F) Cytoplasm positive in plasma cells, with a normal proportion (kappa IHC × 10). (G) Cytoplasm positive in plasma cells, with a normal proportion (lambda IHC × 10).

Bone marrow biopsy demonstrated hypercellularity with marked proliferation of atypical megakaryocytes forming small to large clusters, frequently exhibiting endosteal translocation (Figure 3), a hallmark feature of prefibrotic primary myelofibrosis (pre‐PMF). Granulopoiesis and erythropoiesis were both increased, and no reticulin fibrosis was observed (graded MF‐0). Immunohistochemistry (IHC) confirmed polyclonal plasma cells in normal numbers. The absence of clonal plasma cells in the bone marrow does not preclude the diagnosis of MM [12].

Molecular testing revealed a *JAK2 V617F* mutation, while conventional Giemsa‐banded karyotyping demonstrated a normal female karyotype. A concurrent second primary diagnosis of pre‐PMF was therefore established, meeting the required World Health Organization classification of Haematolymphoid Tumours fifth edition 2022 (WHO Haem 5) major and minor criteria [12].

Prognostic evaluation classified the patient as Intermediate‐1 by the Dynamic International Prognostic Scoring System Plus (DIPSS+; estimated median survival 6.5 years) [15]. Comprehensive genomic profiling was not feasible due to resource constraints, restricting the application of newer mutation‐enhanced prognostic models.

**FIGURE 4:**
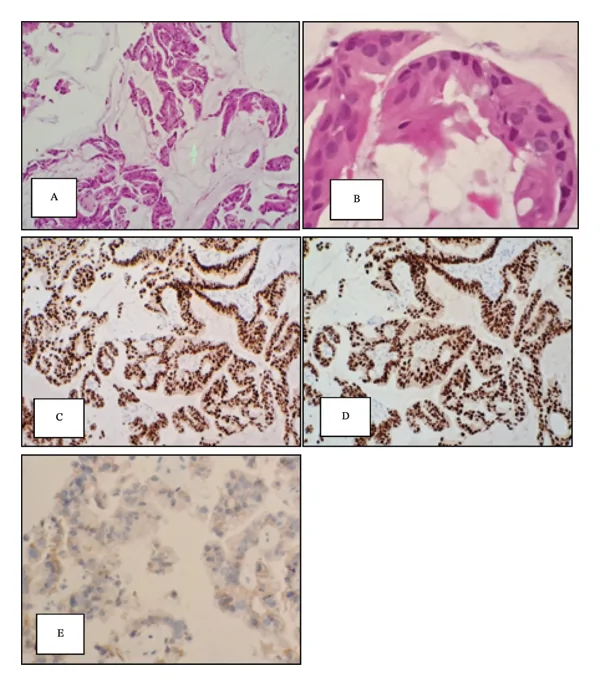
Breast mass core biopsy. (A,B) Malignant epithelial cells with pleomorphic nuclei, forming complex glands, floating in extracellular mucin ((A)H&E × 20; (B) H&E × 40). (C) Oestrogen receptor immunohistochemical stain showed diffuse and strong positivity (ER IHC × 20). (D) Progesterone receptor immunohistochemical stain showed diffuse and strong positivity (ER IHC × 20) (E) Her2 immunohistochemical stain showed 1+ immunoreactivity (> 10% of tumour cells showing faint incomplete membranous staining) (HER2Neu score 1+ negative; HER2 IHC × 40).

During the patient’s hospital stay, a 2‐cm, soft, nontender, mobile mass was detected in the right breast, with no associated skin changes or lymphadenopathy. The mass was congruently identified on CT imaging (Figure 1). Core needle biopsy demonstrated an invasive breast carcinoma with focal mucinous differentiation. IHC revealed oestrogen receptor and progesterone receptor positivity, HER2 negativity and a Ki‐67 proliferation index of 25%, consistent with a luminal B–like profile (Figure 4). As a complete surgical resection was not performed, the relative proportion of the mucinous component could not be determined, limiting definitive classification into pure versus mixed mucinous carcinoma.

Following the diagnosis of breast carcinoma, the patient was commenced on anastrozole, an aromatase inhibitor. On clinical assessment, the breast tumour was staged as T2N0M0. Surgical resection and consideration of adjuvant chemotherapy were deferred. A multidisciplinary team decision was made to prioritise completion of 12 months of therapy for the MM (melphalan and prednisone), with subsequent restaging of the breast carcinoma. At the time of writing, restaging has not yet taken place.

Within 1 month of intramedullary fixation of the femoral fracture, the patient developed local complications, including a left thigh abscess and later a large haematoma, both of which required repeated incision, drainage and secondary wound closure. Following successful rehabilitation, she was discharged home 2 months after the initial fall. At her follow‐up oncology clinic visit, systemic therapy with melphalan and prednisone was initiated. Serum monoclonal protein levels and serum free light chain assays are being monitored as surrogate markers of treatment response.

## 3. Discussion

We describe a highly unusual concurrence of three synchronous primary malignancies—pre‐PMF, MM and mucinous breast carcinoma. While dual primary malignancies are well documented, a triad spanning myeloid, plasma‐cell and epithelial lineages is, to our knowledge, exceedingly rare.

The marked megakaryocytic atypia, persistent leucocytosis and absence of reticulin fibrosis support a diagnosis of pre‐PMF over essential thrombocythaemia, in line with current WHO Haem 5 criteria and expert opinion [12, 16]. Mucinous breast carcinoma can be stratified into pure and mixed subtypes depending on the proportion of mucinous differentiation [17]. Pure mucinous carcinoma generally carries a more favourable prognosis, given lower nodal involvement and fewer high‐risk genomic alterations, but this distinction requires complete excision for confirmation [18–20]. Although the absence of high‐risk cytogenetic abnormalities remains a favourable prognostic feature, R‐ISS Stage 3 represents the highest risk with a 5‐year overall survival of 40% [14]. The outcomes for this stage have improved over recent years with the addition of novel therapies, but disparate accessibility to these therapies remains a problem in resource‐limited settings [21]. There are a number of different regimens which may be used for the management of MM which, in a resource limited setting, depends on the burden of disease, age, transplant candidacy and overall prognosis. A hospital group in Shanghai described the entity ‘macrofocal multiple myeloma’, referring to a subset of MM patients with relatively low bone‐marrow plasma‐cell infiltration but multiple osteolytic lesions, a phenotype associated with more favourable prognosis. Our patient demonstrates features consistent with this description [22, 23].

In patients with concurrent malignancies, individual prognostic models may not adequately capture the overall disease trajectory. A composite risk framework is required, particularly where access to advanced genetic testing, imaging and therapeutics is constrained, further complicating optimal sequential management.

Patients with MPNs have an increased risk of secondary cancers, with up to a 3.0‐fold risk of solid tumours and 3.5‐fold risk of lymphoproliferative neoplasms [24]. Certain mutations (e.g., *U2AF1, DNMT3A, SF3B1* and *EZH2*) are enriched in patients with MPN who develop second malignancies [25]. Many of these overlap with mutations implicated in clonal haematopoiesis (CH), a process also promoted by significant tobacco exposure [26]. CH is increasingly recognised to mediate immune dysregulation and microenvironmental changes that may predispose to secondary solid tumours [27–29].

MPMN may also reflect germline predisposition syndromes. Lynch first proposed this concept in 1978 [30, 31], and germline mutations such as TP53 in Li‐Fraumeni syndrome are now recognised as predisposing to diverse tumour types [30]. Although germline testing was not performed in this patient, the unusual constellation of malignancies raises the possibility of an inherited predisposition.

## 4. Conclusion

The synchronous presentation of multiple unrelated malignancies is exceedingly rare. In this case, the bone lesion might easily have been misinterpreted as metastatic breast carcinoma, while an extramedullary plasmacytoma of the breast could have been considered had the plasmacytoma been identified first. Avoiding diagnostic assumptions and systematically investigating each lesion enabled a timely and comprehensive workup.

In our resource‐limited setting, next‐generation sequencing was unavailable to this patient, restricting both prognostication and the ability to distinguish shared genetic drivers from independent tumour origins.

In conclusion, a multidisciplinary diagnostic approach is essential, particularly in resource‐constrained settings, and clinicians should remain open to the possibility that multiple malignancies presenting simultaneously may represent independent pathologies.

## Author Contributions

**Michael A. Linström**: conceptualisation, writing–original draft preparation (lead), writing–review and editing (lead), supervision and project administration. **Dean C. Mitchell**: investigation, writing–original draft preparation (equal) and writing–review and editing (equal). **Riyaadh Roberts**: writing–original draft preparation (equal) and writing–review and editing (equal). **Robert K. Lohlun**: original draft preparation (equal) and writing–review and editing (equal).

## Funding

This project was supported by the National Health Laboratory Service, Western Cape Department of Health and Stellenbosch University.

## Disclosure

All authors have read and approved the final version of the manuscript. Dr. Michael A. Linström, linstrom@sun.ac.za, had full access to all of the data in this study and takes complete responsibility for the integrity of the data and the accuracy of the data analysis.

## Consent

Informed consent of the patient was obtained and approved by the Stellenbosch University Health Research Ethics Committee with reference number C25/12/047.

## Conflicts of Interest

The authors declare no conflicts of interest.

## Patient Perspective

The patient expressed gratitude to managing clinicians and staff. However, she opted not to provide any detailed feedback on the treatment received.

## Supporting Information

Additional supporting information can be found online in the Supporting Information section.

## Supporting information


**Supporting Information** CARE checklist.

## References

[1] Tanjak P. , Suktitipat B. , Vorasan N. et al., Risks and Cancer Associations of Metachronous and Synchronous Multiple Primary Cancers: A 25-Year Retrospective Study, BMC Cancer. (December 2021) 21, no. 1, 10.1186/s12885-021-08766-9. PMC8461969 34556087

[2] Scott L. C. , Kuo T. M. , Il’yasova D. , and Mobley L. R. , Geospatial Analysis of Multiple Cancers in Individuals in the US, 2004–2014, Annals of Cancer Epidemiology. (March 2021) 5, 10.21037/ace-19-40. PMC8055046 33880445

[3] Pk K. , Prakash G. H. , Kr S. , and Naik R. , Exploring Multiple Primary Malignancies: An Institutional Experience With Dual Malignancy Cases, Asian Pacific Journal of Cancer Care. (November 2024) 9, no. 4, 699–704, 10.31557/apjcc.2024.9.4.699-704.

[4] Zhao J. , Shi L. , Hu W. , Ji M. , Wu J. , and Wu C. , Survival and Clinical Characteristics of Patients With Multiple Primary Cancers: A Hospital-Based Study, International Journal of Clinical and Experimental Medicine. (2019) 12, https://www.ijcem.com/.

[5] Howlader N. , Noone A. , Krapcho M. et al., National Cancer Institute, 2011, SEER Cancer Statistics Review, 1975–2008.

[6] Coyte A. , Morrison D. S. , and McLoone P. , Second Primary Cancer Risk—the Impact of Applying Different Definitions of Multiple Primaries: Results From a Retrospective Population-Based Cancer Registry Study, BMC Cancer. (December 2014) 14, no. 1, 10.1186/1471-2407-14-272. PMC4005906 24742063

[7] Baicry F. , Molinié F. , Plouvier S. et al., What is the Most Appropriate Period to Define Synchronous Cancers?, Cancer Epidemiology. (April 2021) 71, 10.1016/j.canep.2021.101900. 33578073

[8] Hao L. , Zhang L. , Xu C. , Jiang M. , Zhu G. , and Guo J. , Multiple Synchronous Primary Malignant Neoplasms: A Case Report and Literature Review, Oncology Letters. (August 2023) 26, no. 4, 10.3892/ol.2023.14014. PMC10472045 37664660

[9] Katz H. , Jafri H. , Brown L. , and Pacioles T. , Triple Synchronous Primary Malignancies: A Rare Occurrence, BMJ Case Reports. (June 2017) . 10.1136/bcr-2017-219237 PMC5534918 28583923

[10] Oka K. , Nagayama R. , Yonekawa N. , Koyamatsu S. , and Mori N. , A Case Report on a Patient Who Presented With Myelofibrosis, Developed Breast Cancer With Myeloid Metaplasia, and Finally Showed Immature NK-Cell Leukemia or AML Without Maturation With CD56 Expression Transformation in the Pleura With Extramedullary Hematopoiesis: A 25-Year Course, Pathology, Research and Practice. (May 2013) 209, no. 5, 319–322, 10.1016/j.prp.2013.03.007. 23618686

[11] Vennepureddy A. , Motilal Nehru V. , Liu Y. , Mohammad F. , and Atallah J. P. , Synchronous Diagnosis of Multiple Myeloma, Breast Cancer, and Monoclonal B-Cell Lymphocytosis on Initial Presentation, Case Reports in Oncological Medicine. (2016) 2016, 7953745–4, 10.1155/2016/7953745. PMC4877451 27247815

[12] WHO Classification of Tumours Editorial Board , Haematolymphoid Tumors, 2022, 11, 5th edition, International Agency for Research on Cancer.

[13] Rajkumar S. V. , Dimopoulos M. A. , Palumbo A. et al., International Myeloma Working Group Updated Criteria for the Diagnosis of Multiple Myeloma, Lancet Oncology. (November 2014) 15, no. 12, e538–e548, 10.1016/s1470-2045(14)70442-5. 25439696

[14] Palumbo A. , Avet-Loiseau H. , Oliva S. et al., Revised International Staging System for Multiple Myeloma: A Report From International Myeloma Working Group, Journal of Clinical Oncology. (September 2015) 33, no. 26, 2863–2869, 10.1200/jco.2015.61.2267. PMC4846284 26240224

[15] Gangat N. , Caramazza D. , Vaidya R. et al., DIPSS Plus: A Refined Dynamic International Prognostic Scoring System for Primary Myelofibrosis That Incorporates Prognostic Information From Karyotype, Platelet Count, and Transfusion Status, Journal of Clinical Oncology. (February 2011) 29, no. 4, 392–397, 10.1200/jco.2010.32.2446. 21149668

[16] Griesshammer M. , Al-Ali H. K. , Eckardt J. N. et al., How I Diagnose and Treat Patients in the Pre-Fibrotic Phase of Primary Myelofibrosis (Pre-PMF)—Practical Approaches of a German Expert Panel Discussion in 2024, Annals of Hematology. (January 2025) 104, no. 1, 295–306, 10.1007/s00277-025-06191-7. PMC11868337 39888352

[17] WHO Classification of Tumours Editorial Board , Breast Tumours, 2019, 2, 5th edition, International Agency for Research on Cancer.

[18] Hamdy O. , Shetiwy M. , Saber M. M. et al., Mucinous Carcinoma of the Breast: Epidemiological, Clinical, and Prognostic Characteristics; A Single-Center Experience, Discover Oncology. (July 2025) 16, no. 1, 10.1007/s12672-025-03146-2. PMC12287483 40702277

[19] Skotnicki P. , Sas-Korczynska B. , Strzepek L. et al., Pure and Mixed Mucinous Carcinoma of the Breast: A Comparison of Clinical Outcomes and Treatment Results, Breast Journal. (September 2016) 22, no. 5, 529–534, 10.1111/tbj.12621. 27261206

[20] Lu Q. , Zhou H. , Zhang J. et al., Clinicopathological Characteristics and Genomic Profiling of Pure Mucinous Breast Cancer, Breast. (August 2024) 76, 10.1016/j.breast.2024.103760. PMC11231752 38896982

[21] Zanwar S. and Rajkumar S. V. , Current Risk Stratification and Staging of Multiple Myeloma and Related Clonal Plasma Cell Disorders, Leukemia. (November 2025) 39, no. 11, 2610–2617, 10.1038/s41375-025-02654-y. PMC12589131 40702148

[22] Fan J. , Hou J. , Du J. et al., Macrofocal Multple Myeloma Is a Particular Subgroup of Multiple Myeloma, Blood. (December 2015) 126, no. 23, 10.1182/blood.v126.23.1855.1855.

[23] Dou X. , Liu R. , Liu Y. et al., Macrofocal Multiple Myeloma in the Era of Novel Agents in China, Therapeutic Advances in Hematology. (2025) 16, 10.1177/20406207251314696. PMC11783501 39897505

[24] Brabrand M. and Frederiksen H. , Risks of Solid and Lymphoid Malignancies in Patients With Myeloproliferative Neoplasms: Clinical Implications, Cancers (Basel). (October 2020) 12, no. 10, 10.3390/cancers12103061. PMC7589412 33092233

[25] Zhang Y. , Han Y. , Teng G. et al., Incidence and Risk Factors for Second Malignancies Among Patients With Myeloproliferative Neoplasms, Cancer Medicine. (April 2023) 12, no. 8, 9236–9246, 10.1002/cam4.5666. PMC10166886 36727544

[26] Levin M. G. , Nakao T. , Zekavat S. M. et al., Genetics of Smoking and Risk of Clonal Hematopoiesis, Scientific Reports. (May 2022) 12, no. 1, 10.1038/s41598-022-09604-z. PMC9068754 35508625

[27] Pan W. , Zhu S. , Qu K. et al., The DNA Methylcytosine Dioxygenase Tet2 Sustains Immunosuppressive Function of Tumor-Infiltrating Myeloid Cells to Promote Melanoma Progression, Immunity. (August 2017) 47, no. 2, 284–297.e5, 10.1016/j.immuni.2017.07.020. PMC5710009 28813659

[28] Li S. , Feng J. , Wu F. et al., TET2 Promotes Anti‐Tumor Immunity by Governing G‐Mdscs and CD 8 T‐Cell Numbers, EMBO Reports. (October 2020) 21, no. 10. 10.15252/embr.201949425 PMC7534639 32929842

[29] Liu X. , Sato N. , Shimosato Y. et al., Chip-Associated Mutant ASXL1 in Blood Cells Promotes Solid Tumor Progression, Cancer Science. (April 2022) 113, no. 4, 1182–1194, 10.1111/cas.15294. PMC8990791 35133065

[30] Cybulski C. , Nazarali S. , and Narod S. A. , Multiple Primary Cancers as a Guide to Heritability, International Journal of Cancer. (October 2014) 135, no. 8, 1756–1763, 10.1002/ijc.28988. 24945890

[31] Lynch H. T. , Lynch P. M. , and Harris R. E. , Minimal Genetic Findings and Their Cancer Control Implications. A Family With the Cancer Family Syndrome, JAMA. (August 1978) 240, no. 6, 535–538. 671663

