# Supplementary material for: Synchronous Triple Malignancy Presentation of Multiple Myeloma and Prefibrotic Primary Myelofibrosis in a Patient With Mucinous Breast Carcinoma: A Case Report From Rural South Africa
Source: Case Rep Hematol. 2026 Sep 24;2026:7459223. doi: 10.1155/crh/7459223 (PMC13602348; doi:10.1155/crh/7459223)
Supplement: Supplementary file 1 [file CRH-2026-7459223-s001.pdf]

CARE case report guidelines

CARE Checklist of information to include when writing a case report

(cc) BY-NC-ND

Topic	Item	Checklist item description	Reported on Line
Title	1	The diagnosis or intervention of primary focus followed by the words "case report"	1-3
Key Words	2	2 to 5 key words that identify diagnoses or interventions in this case report, including "case report"	52-53
Abstract (no references)	3a	Introduction: What is unique about this case and what does it add to the scientific literature?	56-61
	3b	Main symptoms and/or important clinical findings	64-67
	3c	The main diagnoses, therapeutic interventions, and outcomes	64-73
	3d	Conclusion—What is the main "take-away" lesson(s) from this case?	87-90
Introduction	4	One or two paragraphs summarizing why this case is unique (**may include references**)	96-109
Patient Information	5a	De-identified patient specific information.	112-116
	5b	Primary concerns and symptoms of the patient.	112-116
	5c	Medical, family, and psycho-social history including relevant genetic information	112-116
	5d	Relevant past interventions with outcomes	112-116
Clinical Findings	6	Describe significant physical examination (PE) and important clinical findings.	147-148 174-176
Timeline	7	Historical and current information from this episode of care organized as a timeline	112-205
Diagnostic Assessment	8a	Diagnostic testing (such as PE, laboratory testing, imaging, surveys).	112-184
	8b	Diagnostic challenges (such as access to testing, financial, or cultural)	220-222
	8c	Diagnosis (including other diagnoses considered)	112,135,153
	8d	Prognosis (such as staging in oncology) where applicable	117, 138,171
Therapeutic Intervention	9a	Types of therapeutic intervention (such as pharmacologic, surgical, preventive, self-care)	193 - 205
	9b	Administration of therapeutic intervention (such as dosage, strength, duration)	193-205
	9c	Changes in therapeutic intervention (with rationale)	193-205
Follow-up and Outcomes	10a	Clinician and patient-assessed outcomes (if available)	201-205
	10b	Important follow-up diagnostic and other test results	204-205
	10c	Intervention adherence and tolerability (How was this assessed?)	199-201
	10d	Adverse and unanticipated events	199-201
Discussion	11a	A scientific discussion of the strengths AND limitations associated with this case report	208-244
	11b	Discussion of the relevant medical literature **with references**.	208-244
	11c	The scientific rationale for any conclusions (including assessment of possible causes)	208-244
	11d	The primary "take-away" lessons of this case report (without references) in a one paragraph conclusion	247-257
Patient Perspective	12	The patient should share their perspective in one to two paragraphs on the treatment(s) they received	258-260
Informed Consent	13	Did the patient give informed consent? Please provide if requested	Yes ☑ No ☐
